# The impact of climate on the abundance of *Musca sorbens*, the vector of trachoma

**DOI:** 10.1186/s13071-016-1330-y

**Published:** 2016-01-27

**Authors:** Anita Ramesh, Julie Bristow, Sari Kovats, Steven W. Lindsay, Dominic Haslam, Elena Schmidt, Clare Gilbert

**Affiliations:** Department of Clinical Research, Faculty of Infectious and Tropical Diseases (ITD), London School of Hygiene & Tropical Medicine (LSHTM), Keppel Street, London, UK; Department of Disease Control, ITD, LSHTM, Keppel Street, London, UK; Department of Social and Environmental Health Research, Faculty of Public Health and Policy, LSHTM, Keppel Street, London, UK; School of Biological and Biomedical Sciences, Durham University, Durham, UK; Sightsavers, Haywards Health, West Sussex, UK

**Keywords:** *Musca sorbens*, Diptera, Flies, Climate, Temperature, Rainfall, Humidity, Trachoma, Transmission

## Abstract

**Background:**

To assess the extent to which climate may affect the abundance of *Musca sorbens*, a putative vector of trachoma.

**Data sources:**

Studies were identified by systematically searching online databases including CAB abstracts, Embase, Global Health, Medline, Web of Science and BIOS Online, references from key articles, and the websites of relevant international agencies.

**Methods:**

A systematic literature review was conducted of field and laboratory studies that reported the impact of climate factors (e.g., temperature, humidity) on the synanthropic fly *Musca sorbens*. Data were systematically extracted and studies assessed for quality by two readers. Study results were reported narratively.

**Results:**

A total of 16 studies met the inclusion criteria but only three evaluated associations between climatic/abiotic factors and *M. sorbens*. Limited evidence indicates that *M. sorbens* abundance has an optimal temperature and humidity range. Thirteen studies reported seasonal patterns but no consistent pattern was found between season and the abundance of *M. sorbens*.

**Conclusions:**

The evidence base regarding the effect of climatic factors on *M. sorbens* is limited, so it is difficult to construct a biological model driven by climate for this species. A multivariate statistical approach based on the climate of sites where *M. sorbens* is found may better capture its complex relationship with climatic factors as well as aid in mapping the global range of *M. sorbens*.

**Electronic supplementary material:**

The online version of this article (doi:10.1186/s13071-016-1330-y) contains supplementary material, which is available to authorized users.

## Background

**Fig. 1:**
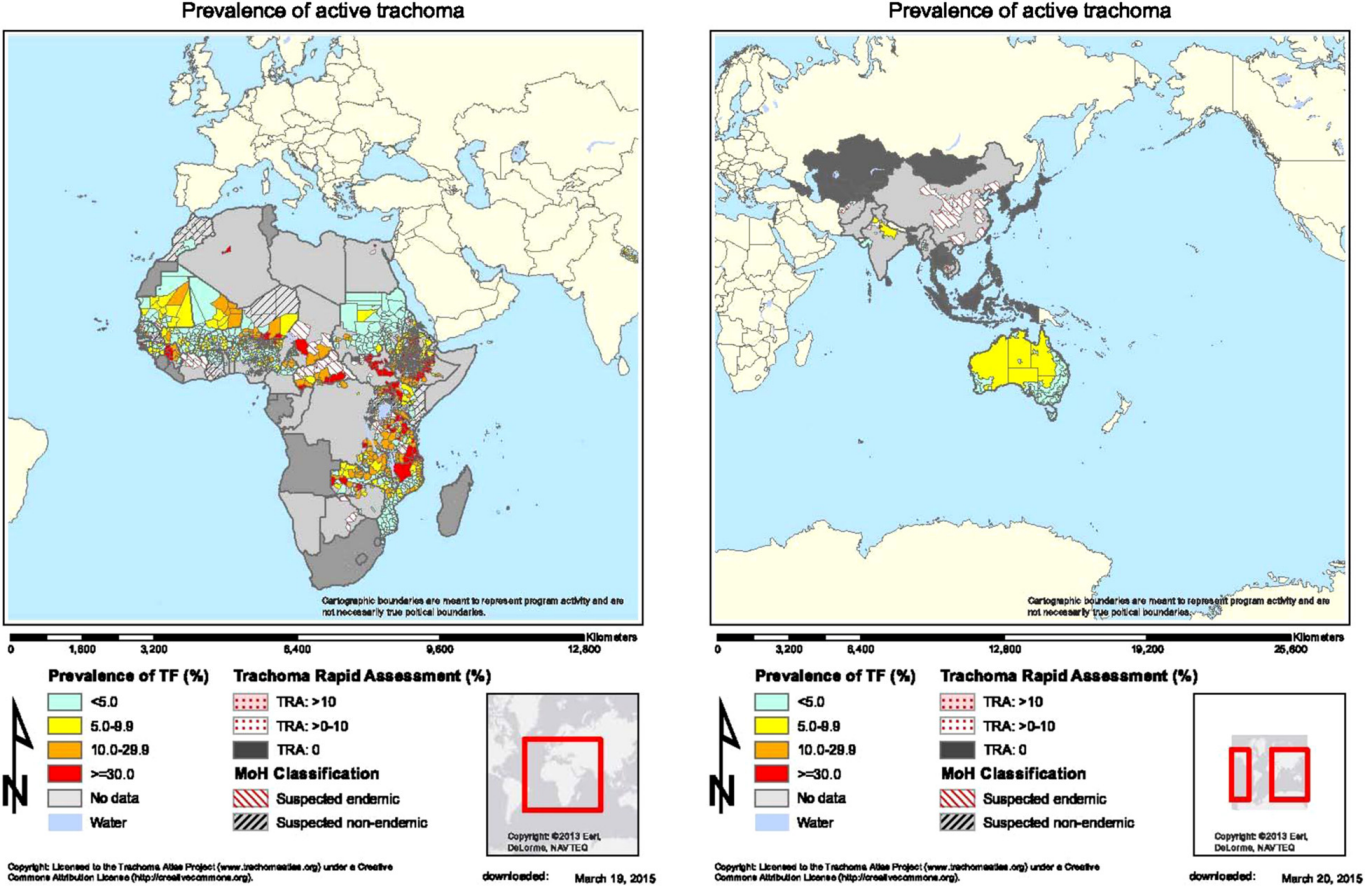
Prevalence of Active Trachoma in Africa, Asia, and the Pacific Islands (Trachoma Atlas, Accessed August 2015)

Trachoma, caused by the bacterium *Chlamydia trachomatis*, is the world’s leading infectious cause of blindness. It is thought to be endemic in 51 countries, primarily in sub-Saharan Africa (Fig. 1) [1]. It is estimated that 232 million people living in trachoma-endemic areas are at risk of the condition; of the over 1.8 million who are visually impaired due to trichiasis (in-turned eyelashes) and its complications, over 500,000 people are irreversibly blind. International efforts are underway to map affected populations and scale-up interventions to eliminate blinding trachoma as a public health problem [2–4].

*Chlamydia trachomatis* can be transmitted directly from person-to-person via contact with infected ocular or nasal secretions, or indirectly from fomites via contact with items such as washcloths and bedding. However, eye-seeking flies – such as the ‘bazaar fly’ *Musca sorbens* (Diptera: Muscidae) in Africa and Asia, the ‘bush fly’ *M. vetustissima in Australia, and* eye gnats *Hippelates* spp*. and Liohippelates* spp*.* in Latin America – have also been implicated in trachoma transmission to varying degrees [5–9]. Female flies feed on human ocular and nasal secretions in order to obtain the nutrients necessary for egg production, so are thought to play a substantial role in fly-related mechanical transmission of trachoma [10]. However, the relative importance of flies in trachoma transmission is likely to vary by geographic location, socio-economic development levels, and time of year. Evidence for a role of *M. sorbens* in the mechanical transmission of trachoma comes from both observational studies in which high fly densities on or around human faces have been associated with trachoma as well as intervention studies in which reductions in *M. sorbens* densities have been associated with a lower incidence of *C. trachomatis* infection [5, 7]. For example, in The Gambia, a randomised controlled trial demonstrated that, insecticide spraying or the provision of pit latrines was associated with a lower incidence of trachoma compared to no intervention [7]. In Tanzania, another trial of insecticide spraying after mass distribution of antibiotics led to a 39 % lower prevalence of trachoma after six months spraying; although this was of borderline significance, after 12 months there was no difference in trachoma prevalence despite consistent reductions in *M. sorbens* abundance [11]. The relative importance of *M. sorbens* as a trachoma vector is likely to vary between geographic locations and change over time.

The geographical distribution of *M. sorbens* overlaps with areas of active transmission of trachoma, as there are areas where *M. sorbens* exists which are not endemic for trachoma (Fig. 1) [12]. A recent systematic literature review of climatic effects on the prevalence, distribution and severity of trachoma concluded that the current distribution of trachoma is associated with higher temperatures, and the prevalence is lower at higher altitudes [13]. This is consistent with what one would expect if flies facilitate transmission [9].

**Fig. 2:**
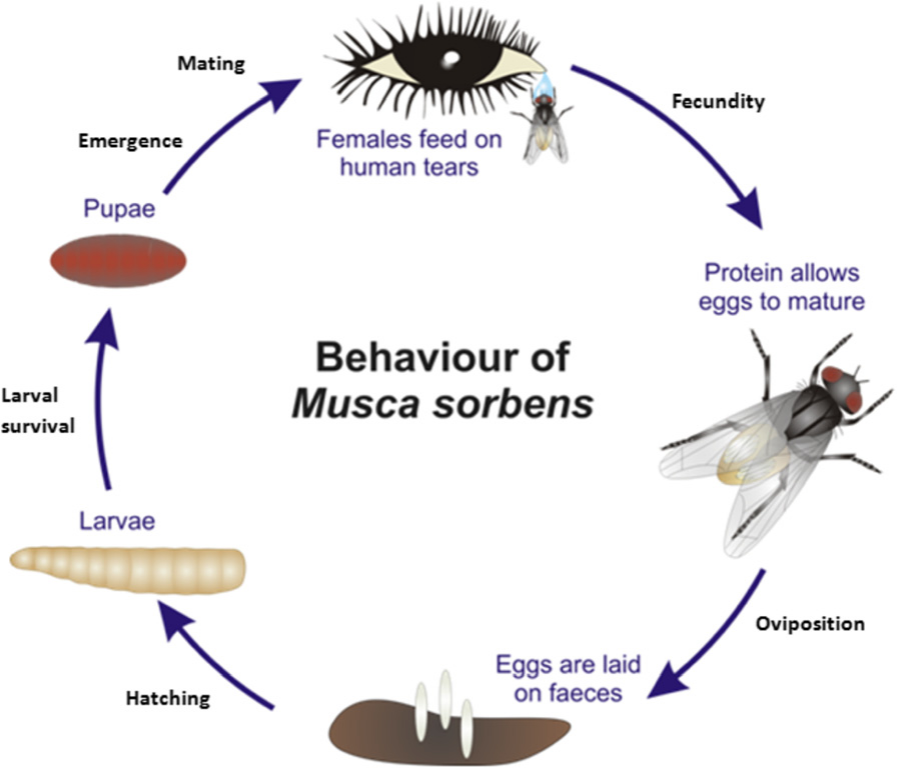
The Life Cycle Of *Musca sorbens,* the Eye-Seeking Fly Implicated in Trachoma Transmission. (Rothamsted Research Visual Communications Unit, Reproduced with Permission)

**Fig. 3:**
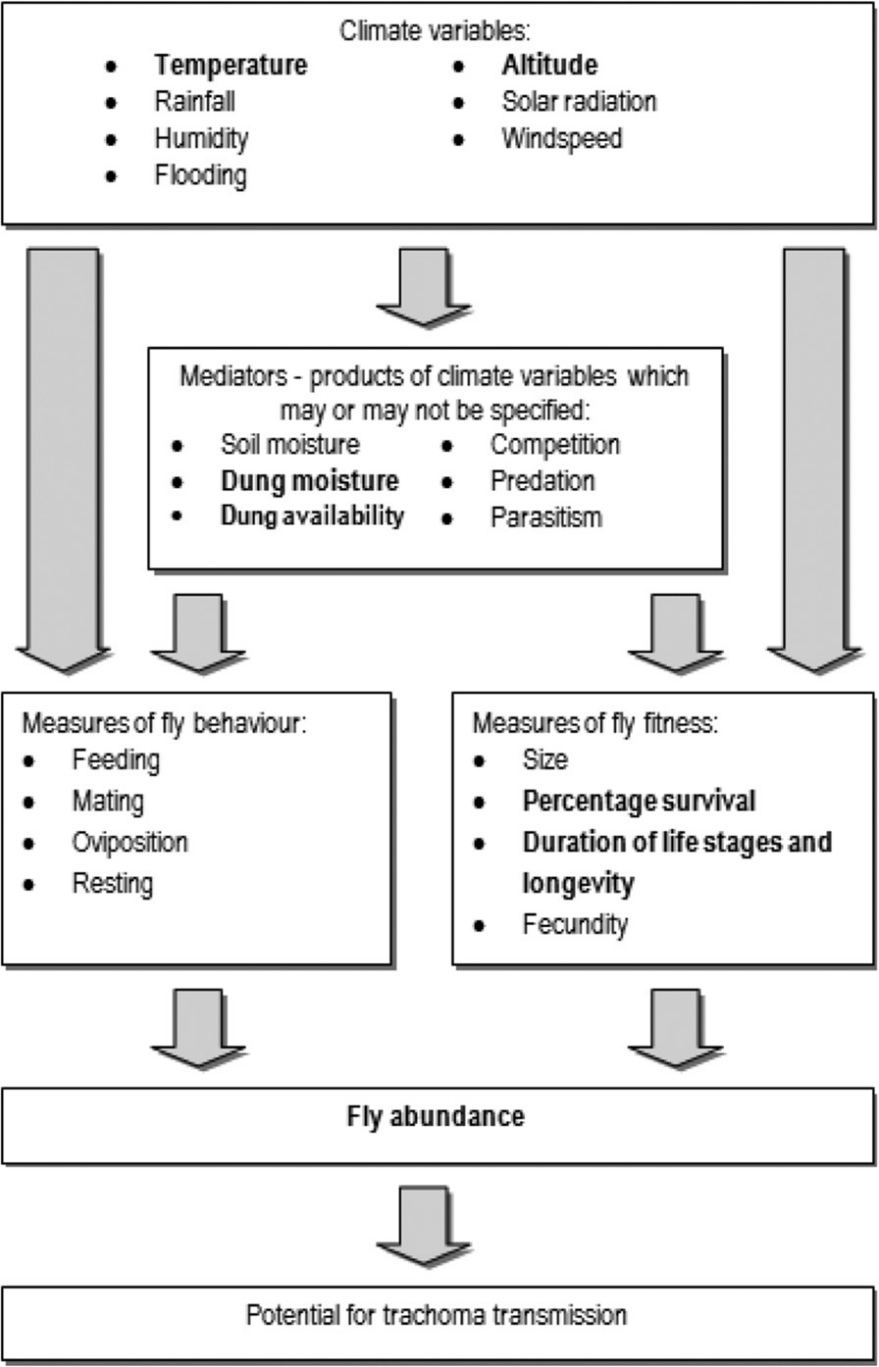
Potential Effects of Climatic Factors and Mediators on Fly Abundance, Fitness, and Trachoma Transmission (Factors Found to be Associated with *M. Sorbens* from Retrieved Papers Marked in Bold)

As has been shown for other insect species, climate affects several stages in the life-cycle of *M. sorbens* (Fig. 2) [14–16]. The vectorial capacity of *M. sorbens* is dependent on the survival of adult flies, the proportion of flies carrying infectious *C. trachomatis,* the number of flies that land on a face, and the proportion of these fly-human contacts that leads to infection. The development, survival and activity of flies is likely to be associated with ambient temperature and humidity. There is also an optimum range of temperature and humidity for the fly, above and below which the fly will fail to thrive (Fig. 3) [17–20]. Climate may also influence the abundance of *M. sorbens* through effects on environmental mediators, such as access to faeces for egg production, breeding, and maturation. Adult females preferentially oviposit on human faeces close to the ground, and the number and size of emerging offspring are greater for human than herbivore faeces, suggesting that human faeces provides the optimal conditions for fly development [6, 8, 21, 22]. High temperatures combined with low humidity could dry out faeces or lead to crust formation on faeces, with the potential to limit adult emergence [23]. Climatic conditions that favour the growth of dung beetle populations, such as warm and wet weather, could lead to a reduction in *M. sorbens*; since dung beetles can remove dung from the soil surface within several hours [24].

The purpose of this study was to review the availability and quality of the evidence for the impact of climate factors on *M. sorbens* abundance, and to identify gaps in the literature on this under-researched vector.

## Methods

A systematic literature review was conducted to evaluate the evidence for an association between climatic factors and the abundance of *M. sorbens*. Additional file 1: Table S1 describes the search terms, key words, and MESH headings used. The following outcomes in relation to *M. sorbens* were included: 1) abundance of adult flies*;* 2) fitness *(*size, life span/longevity, basic reproductive number and parity); 3) adult fly behaviour (e.g., daily activity times, feeding, mating, and oviposition); and 4) life cycle (i.e., development and maturation of eggs, pupae, and larvae). Climate-related exposure terms included: meteorological factors (temperature, rainfall, humidity, sunlight), weather events (e.g., drought, floods), and the related abiotic exposures for laboratory based studies (e.g., temperature, humidity, radiation). As *M. sorbens* is largely exophilic, fly count data from catches made outdoors are reported [25].

### Inclusion criteria

Peer reviewed journal articles published between 1 January 1950 and 1 August 2013; field studies (observational, cross-sectional, intervention or spatial) regarding the effect of climate factors on the abundance of *M. sorbens*; laboratory studies reporting an association between temperature, humidity, or other abiotic factors and *M. sorbens* outcomes, and studies describing seasonal patterns of fly counts.

### Exclusion criteria

Review articles and conference abstracts.

There were no restrictions on geographic location of study, or language of journal publication.

Structured searches were conducted in the following standardized electronic databases: CAB, abstracts, Embase, Global Health, Medline, Web of Science and BIOS Online. Reference lists of selected articles were examined to find any other relevant papers. Limited searches were conducted among the following expert sources: World Health Organization (WHO) Special Programme for Research and Training in Tropical Diseases (TDR): http://www.who.int/tdr/en/; United Nations Children’s Fund: http://www.unicef.org/; The Carter Center: http://www.cartercenter.org/; The International Trachoma Initiative (ITI): http://trachoma.org/; Tropical Diseases Bulletin from 1900 to 2010.

### Data extraction and analysis

The review was conducted by two readers (JB and AR). An initial search was conducted of each database for all fly species implicated in trachoma transmission (see Additional file 1: Table S1). Titles were scanned to ensure that articles related to at least some aspect of the study question. Titles and abstracts were then reviewed to exclude papers that did not meet the inclusion criteria. Full texts of papers provisionally included were then obtained and inclusion criteria checked by the second reader. Data were then extracted using a standard format to collect data on study setting, design and target population, the nature of the study, climate factors and how they were measured, fly outcomes and how they were measured, and whether there were any statistical tests of association.

### Quality assessment

A modified version of a quality assessment tool developed for quantitative studies was used to assess the quality of each study [26]. Each study was assessed as providing strong, moderate or weak evidence depending on methods of sampling, duration of sample, sample size, minimization of bias, control of confounding, and exposure assessment. Studies that met the criteria were assessed as having high, moderate, or poor quality. Studies graded to be of poor quality included studies where only descriptive data on fly abundance were presented.

It was anticipated that heterogeneity in study designs and exposure measurements would preclude a meta-analysis; thus, findings were compiled and reported using a narrative synthesis.

## Results

**Fig. 4:**
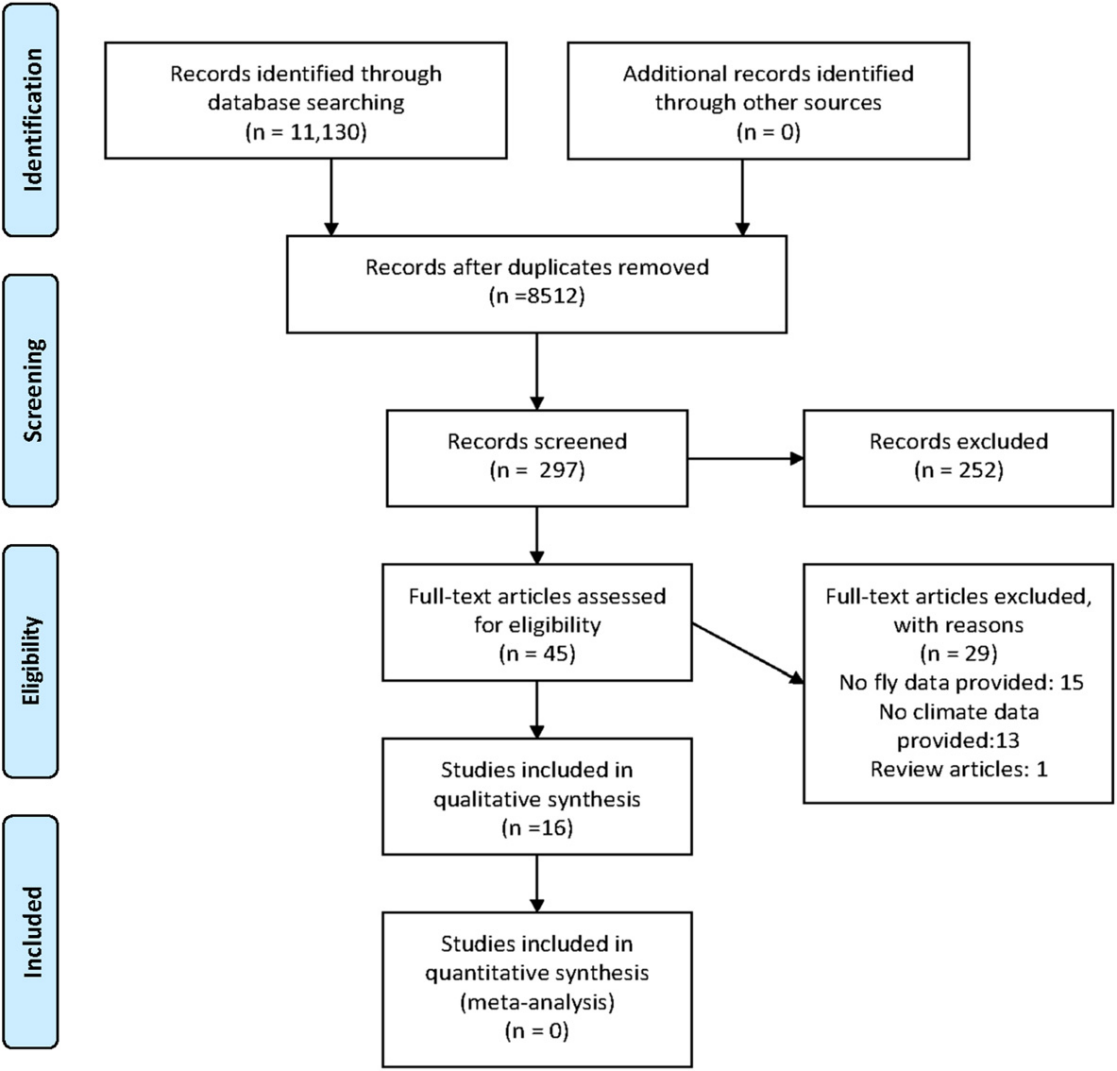
PRISMA Process of Paper Selection

From 11,130 that were initially recovered, a total of 16 studies met the criteria for our review (Fig. 4). Of the original 11,130 studies initially recovered, 10,833 were removed as duplicates or completely non-topical. Of remaining 297 abstracts screened for full review, 252 studies were excluded because they lacked data on flies, climate variables, or both, and one article was not based on primary data.

The 16 studies included in this review were heterogeneous with respect to outcome measures and methods used. Most studies were from previous decades, and were undertaken in a wide range of locations around the world. Most were field studies, and only one laboratory-based study was identified. Table 1 describes the two studies that were assessed to be of highest quality, Table 2 details the one study assessed to be of moderate quality, while Table 3 provides information on the thirteen studies deemed to be of low quality while still providing some possible insights related to seasonal effects on flies.

**Table 1:** Effects of Climatic Factors on the Abundance of *M. sorbens, High Quality Studies*

Authors, Study Type, and Location	Climate Exposure(s), Measure(s)	Fly Outcomes	Methods	Results (Prevalence/Odds Ratio, 95 % Confidence Interval (CI)) *Where noted CI = Credibility Interval*)	Conclusions
Authors: Toyama & Ikeda, 1981 [23]	Tempera-ture: 12 mm below surface of dung pat	Adult: abundance	Method: Flies trapped for 5 min using cone traps baited with fresh cow dung pats; Frequency: Bi-weekly for 11 months then weekly for an additional 14 months.	Adult *M. sorbens* is significantly more abundant in summer than winter (*p* < 0.001).	Direct effects: Summer (i.e., higher temperature) is significantly associated with greater abundance of all life stages of *Musca sorbens*.
Type: Field study	Season: Summer (May - October) and winter (November – April); year unstated				Indirect effects: Crusts on dung pats at higher summer temperatures reduce breeding potential of parasites which predate developing *M sorbens*. Greater abundance of *M sorbens* in summer may therefore reflect lower predation i.e., an indirect effect of temperature.
Location: Dairy farm, Oahu, Hawaii, USA		Egg and larval abundance	Method: Dung pat infestation rates: 20 randomly selected dung pats were examined for eggs and 20 for larvae. Frequency: Weekly between 10:00–20:00 h, for 15 months.	Significantly more dung pats with eggs and larvae found in summer (*p* < 0.001). Dung pat temperatures were higher than ambient temperatures only in summer (ambient 29.16 ± 0.80C, dung pat 33.36 ± 1.14C; *p* < 0.001). Dung pats developed thick, hard crusts during summer, but were thin and softened each night from moisture during winter. There were significantly more flies in unsoftened than softened pats (*p* < 0.001), and higher rates of parasitism of larvae in winter than summer (14 %, ns).	
		Larval abundance	Method: Dung pat larval examinations: 4 dung pats more than 3 days old were collected and examined. Frequency Monthly for 13 months.		
Authors: Taye et al., 2007 [9]	t, November 2003 and February	Adult abundance	Method: Human landing catches on young children: counts of fly-eye contacts over 10 min outdoors, a short break, then 10 min indoors between 8:30 and 13:00. Frequency: Bi-weekly for 11 months then weekly for an additional 14 months.	Almost all eye-seeking *M. sorbens* were captured outdoors at low and at medium altitudes [99.3 %; 95 % confidence interval (CI) 99.1 %–99.5 %; *p* < 0.001].	Direct effects: Medium altitude is significantly associated with greater abundance of adult *M. sorbens.*
Type: Field study	Altitude: <2000 m				
	2200–2500 m				
	>3000 m				
Location: 12 villages in Gurage zone, Ethiopia.	Season: May, August, November 2003 and February 2004	Adult abundance	Method: Modified WHO-exit traps baited with beef 10 traps over 25 h, 1.5 m above ground in playground, plantation, pits, shade, and indoors Frequency: Weekly between the hours of 10:00 and 20:00, for 15 months.	*M. sorbens* numbers declined with increasing altitude - of the 3465 *M. sorbens* trapped, 55.6 % and 44.3 % were collected in the low- and mid-altitude villages, respectively, but only 0.1 % (*p* = 0.001) came from villages at higher altitudes.

**Table 2:** Effects of Climatic Factors on the Abundance of *M. sorbens, Moderate Quality Studies (from Hafez and Attia, 1958* [22])

Authors, Study Type, and Location	Climate Exposure(s), Measure(s)	Fly Outcomes	Methods	Results (Prevalence / Odds Ratio, 95 % Confidence Interval (CI)) *Where noted CI = Credibility Interval*)	Conclusions
Authors: Hafez and Attia, 1958 [22]	Temperature	Egg incubation period	Humidity maintained at 100 % RH. Between 53 and 60 eggs were studied at 5.5 °C, 16 °C, 24 °C, 28 °C, 32 °C, 36 °C, 40 °C and 43 °C.	Eggs failed to hatch at 5.5 °C or 43 °C . Eggs took an average of 5.28 h to hatch at 40 °C and 25.3 h at 16 °C.	Within temperature limits that permitted hatching, incubation period was inversely proportional to temperature. Optimal hatching occurred at 28C. No statistical measures.
Type: Lab study					
Location: Taliba, Egypt.		Egg hatching percentage	Eggs were fertile and secured from copulated females.	At 16 °C 95 % of eggs hatched. At 28 °C 100 % of eggs hatched. At 36 °C 90 % of eggs At 40 °C 65 % of eggs hatched.	
		Larvae Duration of larval period	Larvae were reared on 15 g milk/bran larval diet, 10 eggs in each 10 cc tube. They were then emptied onto a drier pupation medium.	At 16 °C the mean duration of the larval period was 268.95 ± 0.94 h. At 40C it was 73.05 ± 0.71 h.	Larval period duration was inversely proportional to temperature. Failure to provide drier pupation medium prolonged the larval period duration by several days. No statistical measures.
		Pupae Duration of pupal period	Humidity maintained at 70 % RH. 42–58 pupae were studied at temperatures of 16 °C, 24 °C, 28 °C, 32 °C, 36 °C and 40 °C.	At 16 °C the mean duration of the pupal period was 14.9 ± 0.2 h. At 36 °C it was 3.86 ± 0.02 h.	Pupal period duration was inversely proportional to temperature.
		Adult Percentage adult emergence	Humidity maintained at 70 % RH. 90–115 pupae were studied at temperatures of 16 °C, 24 °C, 28 °C, 32 °C, 36 °C and 40 °C.	66.6 % of adults emerged at 16 °C. 94.7 % emerged at 24 °C and 28 °C.90 % emerged at 32 °C. None emerged at 40 °C.	Maximum emergence occurred between 24C and 28C. 40C temperatures prevent emergence. No statistical measures.
Authors: Hafez and Attia, 1958 [22]	Relative Humidity	Egg incubation period	Temperature maintained at 31–32 °C. No information given on numbers used.	At 100 % humidity eggs took 6.3–6.6 h to hatch. At 95 % humidity eggs took 6.6–7.1 h to hatch. At 90 % humidity eggs took 7.1–6.6 h to hatch. At 85 % humidity no eggs hatched.	Eggs did not hatch at humidities below 85 %, and took longer to hatch at lower humidities. Fewer eggs hatched at lower humidities. Low humidities damaged eggs through dehydration, causing shrinkage. No statistical measures.
Type: Lab study					
Location: Taliba, Egypt.		Egg hatching percentage	Temperature maintained at 31–32 °C. No information given on numbers used.	At 100 % humidity 100 % of eggs hatched. At 90 % humidity 58 % of eggs hatched. At 85 % humidity no eggs hatched.	
		Egg structure	Fertile eggs laid within 15 min of the start of the experiment were placed in a desiccator.	Egg length/width: When RH was increased from 0 to 100 % length increased from 1.369 mm to 1.465 mm (average elongation 7 %) and width increased from 0.298 mm to 0.312 mm (average increase in width of 4.7 %).	
				Egg weight (water loss): When exposed to 30 % RH, eggs lost 30 % of their weight in first 60 min (20 % in first 30 min, 10 % in last 30 min). Water loss decreased with increasing exposure to low RH.	
		Pupae Duration of pupal period	Temperatures of 28^0^ and 36^0^ %.	The pupal phase lasted 106–118 h at 28 °C regardless of humidity, and between 90 and 93 h at 36 °C regardless of humidity.	Relative humidity has no significant effect on duration of the pupal phase.
			No information given on numbers used.		

Three studies examined associations between distribution of flies and climatic factors [9, 22, 23].

### Temperature

Toyama and Ikeda (1981) studied the role of climate factors on flies in a field study in Hawaii (Table 1) [23]. Summer season was significantly associated with greater abundance of all life stages of *M. sorbens*. The study also found that the crusts on dung pats at higher summer temperatures reduced predation, most likely by *Labidura riparia*, an earwig, which was unable to penetrate the pats to hunt fly larvae. In contrast, during the winter months, no such crust formed on the pats, and the earwigs depressed the population of *M. sorbens*.

### Altitude

Taye et al. (2007) reported the abundance of *M. sorbens* caught on children’s faces (indoors and outdoors) and in traps in Ethiopia, during four different seasons and at three altitudes, the latter being a proxy measure of ambient temperature (Table 1) [9]. Over 99 % of *M. sorbens* were caught outdoors and only 0.7 % of *M sorbens were* caught on children living above 3,000 m. At altitudes <2000 m there was less seasonal variation in the median number of *M. sorbens* caught per child, ranging from 7/child in the dry, winter season to 13/child in the wet, summer season. A different seasonal pattern and range was reported at higher altitude (2200–2,500 m) with the number of *M. sorbens* caught on children’s faces ranging from 2.5/child in the wet, summer season to 23/child in the dry, winter season. Findings were similar from *M. sorbens* caught in beef-baited traps. At <2000 m there were higher catches in all four seasons with not much seasonal variation (402 in the dry winter compared with 611 in semi-dry spring) compared with higher altitude (2200–2500 m) where the lowest counts were in the wet summer season (11 *M. sorbens*) and highest in the semi-wet autumn season (305 *M. sorbens*). The authors comment that at altitudes <2000 m the annual temperature ranges from 23 °C in the wet season to 36 °C in the dry season and from 5 to 18 °C above 3,000 m but details were not published. There were no tests of association between season, altitude and *M. sorbens* counts.

### Relative humidity

**Fig. 5:**
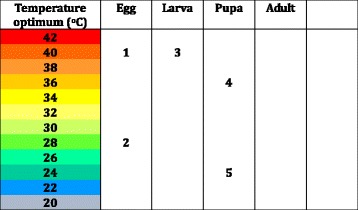
Temperature Optima for Different Life Stages of *M. sorbens, High and Moderate Quality Studies*. 1. Shortest egg hatching time, Hafez & Attia 1958 [21, 22]. 2. Highest percentage egg hatching, Hafez & Attia 1958 [21, 22]. 3. Shortest larval period, Hafez & Attia 1958 [21, 22]. 4. Shortest duration of pupal period, Hafez & Attia 1958 [21, 22]. 5. Highest percentage of pupal hatching, Hafex & Attia 1958 [21, 22]

**Fig. 6:**
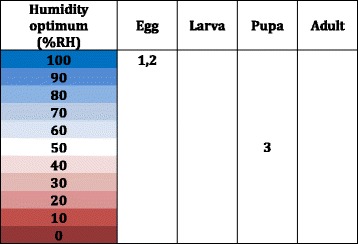
Humidity Optima for Different Life Stages of *M. sorbens, High and Moderate Quality Studies*. 1. Shortest egg hatching time, Hafez & Attia 1958 [21, 22]. 2. Highest percentage egg hatching, Hafez & Attia 1958 [21, 22]. 3. Pupal phase duration was independent of humidity at humidities studied by Hafez & Attia 1958 [21, 22]

Hafez and Attia (1958) explored the impact of relative humidity and temperature on different aspects of *M. sorbens* life cycle (egg incubation period, egg hatching rate, duration of the larval and pupal periods, and egg structure (humidity only) in laboratory studies (Table 2) [22]. At 100 % humidity, egg hatching occurred across a wide temperature range, with the optimal temperature for hatching being 28 °C. At 70 % humidity, adult emergence was maximal between 24 and 28 °C, but did not occur above 40 °C. When temperature was maintained at 31–32 °C, eggs did not hatch at humidities below 80 %, but 100 % of eggs hatched at 100 % humidity (Fig. 5 and 6). None of these associations were assessed statistically.

The three higher quality papers demonstrate that, amongst the climatic factors studied, temperature appears to be the most strongly associated with *M. sorbens* abundance. Consistent with other Muscids, like *M. domestica* and *M. autumnalis*, *M. sorbens* have an optimum temperature for survival and behaviour, after which increasing temperatures are associated with a decrease in fly abundance [27–29]. Importantly, each species have different optimal conditions for their survival. The paper by Toyama et al. presents a plausible explanation why *M. sorbens* no longer thrive at high temperatures (e.g., high temperature leading to crust formation on dung pats, thereby hindering fly emergence) [23].

The remaining 13 studies are described in Table 3 [21, 30–40]. These studies are mostly descriptive in nature, and report the relative abundance of *M. sorbens* in relation to other fly species, by season or month. Additional information on the climate for each location was obtained to see if there was consistency across the climate factors that described the peak fly season.

**Table 3:** The Effect of Climatic Factors and Seasonality on *M. sorbens A*bundance. *Low Quality Studies*

Reference and Study Location	Fly Outcome	Methods	Results	Climatic or Seasonal Effects
Author(s): Ponghis (1957) [36]	Adult abundance	Collection type: Traps (type unstated)	Mean monthly catches were highest in June and October, when *M. sorbens* comprised 1 % and 30 %, respectively, in relation to *M domestica*. From databases: June: average max temp 24 °C, Oct 25 °C; June av ppt 1 mm; Oct 26 mm).	*M. sorbens* abundance was greatest in spring and autumn (i.e., just before and after the hottest, driest months). Heat and dryness had an unfavourable effect on fly multiplication. No statistical measures.
Location: 2 villages, Southern Morocco		Frequency: 1 x / week, June – December (1956)		
		Other details: Traps baited with human faeces, fruit, or vegetables. Precautions taken to eliminate effects of secondary factors (e.g., timing of trap deployment).		
Author(s): Miranpuri and Lahkar (1980) [35]	Adult abundance	Collection type: Fly nets	*M. sorbens* numbers appear to peak between July - October, with the highest peaks in August (end of hot- wet season) and September (start of dry-hot season). From databases: April: Av max temp: 28 °C and av ppt 3 mm. June Av max temp: 35 °C and av ppt 0 mm August Av max temp 31 °C, av ppt: 462 mm.	*M. sorbens* abundance was greatest towards the end of the hot, wet season and the beginning of the dry-hot season. No statistical measures.
		Frequency: 4 x / month, 2 h twice per day (8–10 am; 13–15 pm); 1974–5 (exact period unstated).		
Location: Cattle sheds, Assam, India				
Author(s): Amin et al. (1998) [30]	Adult abundance	Collection type: Cone traps	Relative distribution of fly species by season. Mean monthly catches were highest in April, February and August, and lowest in November, September and July. From databases: April: Av max temp: 28 °C and av ppt 3 mm. June Av max temp: 35 °C and av ppt 0 mm	*M. sorbens* accounted for 8.5 % of all flies caught throughout the year. *M sorbens* demonstrated two seasonal peaks, in April and June, when it was the second or third most populous species caught. No statistical measures.
		Frequency: 1 x / month, December 1994 – November 1995		
Location: Al Amargh, Cairo (outskirts), Egypt.		Other details: Traps baited with meat, fish, or liver “near fly breeding sites”.		
Author(s): Khan et al. (1965) [32]	Adult abundance	Collection type: Method not stated	*M. sorbens* is “essentially a fly of the plains, being entirely absent at higher elevations”. In Chandigargh (387.5 m), for instance, it comprised over 78 % of flies caught whereas in Simla (2202 m) none were caught.	*M. sorbens* was not found at high altitude (>2000 m), a proxy for low temperature. No statistical measures.
		Frequency: 3 x / day, 30 min each (duration not provided)		
Location: India (National Study)		Other details: Altitude (m) per location: 11 m (Bombay) - >2000 m (Simla).		
Author(s): Rechav (1989) [37]	Adult abundance	Collection type: Hand net	*M. sorbens* accounted for 1.6 % of all flies caught.	*M. sorbens* abundance was greatest in late winter / early spring, but were not abundant at any time in this study. No statistical measures.
		Frequency: 1 x month, collected between 11 am – 12 pm each day over 14 months (years unstated).	*M. sorbens* numbers peak in August/September. None were caught in many months outside this season. From databases: August av max temp: 22 °C and av ppt 5 mm; September av max temp: 25 °C and av ppt 20 mm	
Location: Cattle sheds, Transvaal, South Africa		Other details: 10 ml of fresh blood was poured on 12 cows.		
Author(s): Sukhova (1963) [38]	Adult abundance	Collection type: Fly traps	*M sorbens* displayed two seasonal peaks (summer, autumn). In the north, *M sorbens* peaked in summer only. From databases: Hottest summer months June to August: Av max temp: 35–36 °C and av ppt 33–38 mm.	*No numbers only figures provided.* No statistical measures.
Location: South, Southwest and Northern Turkey.		Frequency: Time / duration not provided; day and night trapping reported.		
Author(s): Tawfik (1969) [39] N	Adult abundance	Collection type: Wire mesh cone traps	Daily temperature: *M. sorbens* numbers were highest on days with average daily temperatures of 26–26.7 °C; in summer, *M. sorbens* numbers peaked between 6 and 7 am, and 5 and 7 pm whereas in winter, *M sorbens* numbers peaked at 8 am then rose with temperature to peak at 12 pm.	*M. sorbens* abundance was greatest at higher temperatures i.e., 22–23C. No statistical measures.
Location: Cairo (outskirts), Egypt.		Frequency: 2 x / month, hourly catches on two consecutive days		
		Other details: Traps baited with rotten fish.		
			Season/temperature/humidity: *M sorbens* numbers were highest in spring (April), corresponding to mean temperature of 22.9 °C and RH 38 %; second peak in autumn (October) with temperature 22.8 °C and RH of 56 %.	
Author(s): Hafez and Attia (1958) [21]	Adult abundance	Collection type: method not reported	Mean monthly catches were highest in April, February and August, and lowest in November, September and July.	*M. sorbens* abundance was greatest in spring and summer and were lowest in winter. No statistical measures.
		Frequency: 1 x / week, entire year (1957)		
Location: Cairo (outskirts), Egypt.		Other details: Flies ‘attracted to children’s’ eyes’ were collected.		
Author(s): Koe (1975)	Adult abundance	Collection type: Traps (method not reported)	*M. sorbens* numbers appear to peak in August, and are lowest in April. It was the fourth most populous species caught.	*M. sorbens* abundance was greatest in late summer. No statistical measures.
Location: 1 county, Central China		Frequency: 1 x / month, April - November 1963		
		Other details: Fermented bran and sugar bait-trap method.		
Author(s): Wang at al. (2000) [40]	Adult abundance	Collection type: Cylindrical traps	*M. sorbens* was the first most populous species caught in 1998 and the second in 1999.	*M. sorbens* abundance was greatest summer and autumn. No statistical measures.
		Frequency: 3 x / month, June to November (1998) and March to November (1999), between 8 am to 4 pm		
Location: Jinhua City, Zhejiang Province, Southern China		Other details: traps baited with fish, fermented bean curd and sugar.	Mean monthly catches were highest in October1998 and August 1999.	
Author(s): Liu at al. (2010) [34]	Adult abundance	Collection type: Traps (method not reported)	Mean monthly catches appear to peak from June to August, with the highest peaks in July.	*M. sorbens* was the most populous species caught accounting for 48.7 % of all flies caught. No statistical measures.
Location: Ankang City, Shaanxi Province, Western China		Frequency: Time and duration of catches unknown, January - December 2008.		
Author(s): He at al. (2011) [31]	Adult abundance	Collection type: Conical traps	*M. sorbens* accounted for 1.97 % of all flies caught, but peaked in November and January.	*M. sorbens* numbers appeared to peak in November, and were lowest in January. No statistical measures.
Location: Qingyuan City, Guangdong Province, Southern China		Frequency: 1 x / month, September 2005 - August 2010		
		Other details: Traps baited with vinegar and sugar; deployed 9 am–12 pm and 3–4 pm.		

## Discussion and conclusion

This review of potential associations between climatic factors and *M. sorbens* was conducted in order to provide evidence on the climatic boundaries beyond which *M. sorbens* is unlikely to be a vector of trachoma. Outside these climatic constraints, transmission of *C. trachomatis* is more likely to be through person-to-person contact rather than via flies. Consequently, in such conditions, interventions should focus on facial cleanliness, other behaviours, and reductions in overcrowding. It was envisioned that insights gained could also assist in planning future trachoma control strategies in the face of possible global climate change. However, this systematic literature review indicates that evidence for the effect of climate factors on the abundance and seasonal activity of *M. sorbens* is very limited.

One reason for the lack of evidence regarding climatic factors which affect *M. sorbens* is that the fly has only recently been implicated as an important route of transmission in trachoma [7]. In contrast, the vectors of many other infectious diseases (e.g., malaria, dengue, leishmaniasis and trypanosomiasis) have been studied for years in order to improve vector control methods. Maps of disease vectors generally lag behind those of the diseases themselves, although the recently-produced Malaria Atlas provides a comprehensive, well-funded project mapping the distribution of the forty one dominant malaria vector species [41]. The effect of climatic factors on the biology of malaria vectors has also been studied, allowing for the development of models that forecast possible effects of climate change on the distribution of malaria vectors and hence vectorial capacity [42, 43]. Whilst the Trachoma Atlas is currently being compiled to map the distribution and endemicity of trachoma, at present there is no equivalent map of the distribution of *M. sorbens* and the effects of climatic factors on the abundance and distribution of this fly remain poorly understood in comparison to the state of knowledge on malaria vectors [3].

Although there is still some uncertainty about the relative importance of transmission by *M. sorbens*, results of this review support the implementation of the SAFE (S = surgery, A = mass antibiotic distribution, F = facial cleanliness to reduce ocular secretions and, E = environmental hygiene) strategy, which is being led and scaled-up by the WHO Alliance for the Global Elimination of Blinding Trachoma (*GET 2020*) with the aim of eliminating blinding trachoma by the year 2020 [32]. In particular, the aim of the ‘E’ component of the strategy is to reduce *C. trachomatis* transmission by means of environmental improvements such as water and sanitation; increased access to water could improve hygiene, while reductions in open defecation – which provides ample, open breeding sites for *M. sorbens* – could result in reductions in trachoma [6, 44].

This systematic review had several limitations. First, given the dearth of moderate and high quality evidence related to the effects of climate factors on *M. sorbens*, it was difficult to interpret the results of the very limited evidence base. The sixteen studies included in this review employed a wide variety of designs, methods, and analytical tools. Only one study provided evidence from a laboratory setting, and this study was conducted nearly 60 years ago, when methods and analytical techniques were less advanced than at present [22]. The highest quality papers were both conducted within the last 30 years and used statistical methods to provide evidence of the strength of associations [9, 23]. Unfortunately most studies reviewed did not provide statistical evidence. None of the studies used robust designs, and none provided details on all of the entomological methods used (e.g., trap designs, speciation methods, etc.). These limitations mean that most studies were categorized as being of poor quality and the results were difficult to interpret.

There is a need for more evidence on the relationship between climatic factors and *M. sorbens* distribution and abundance which could be used to guide whether the F and/or E elements of the SAFE strategy need to be prioritized. For example, interventions in locations where climatic conditions promote *M. sorbens* abundance all the year round need to focus on all elements of the SAFE strategy. In locations where climatic factors promote seasonal variation in the abundance of *M sorbens* targeted vector control could be considered. In locations where climatic factors are not conducive to *M. sorbens* the F elements need to be promoted to reduce transmission via fomites.

This review found a dearth of data linking climate variables with important life-history traits of *M. sorbens*. Such data are typically used to construct mathematical models that allow investigators to explore the impact of climate change on the risk of vector-borne diseases. One alternative approach that is used commonly by biologists where detailed data on a species is lacking is to produce maps based on the climate at sites where a species was recorded i.e., presence only data [45]. Machine-learning techniques and community models, that are well suited to sparse occurrence data, are then used to map a species’ distribution based on climate factors. The statistical relationship that best captures the climate envelope for the fly can be used to explore future distributions when it is linked to future climate change scenarios [46]. Such an approach would require collating data on the distribution of this species from museum collections, record lists and active surveys.

An alternative approach is to build mathematical models that describe the relationship between the life stages of *M. sorbens* and climate based on what is known for other Muscids. For example the relationship between climate and the bionomics of the house fly, *M. domestica*, and the face fly, *M. autumnalis*, have been described in more detail [27–29]. These models could be used to help produce models for M. sorbens where there are few data.

Overall, *M. sorbens* is adapted to hot tropical climates and can withstand highly desiccating environments. It breeds most actively in hot humid conditions, but this relationship is likely to be non-linear with extremely high temperatures reducing fly survival. Indirect effects of climate on fly abundance are difficult to predict, but higher temperatures and low humidity may create a hard crust on stools harbouring immature flies, restricting the emergence of the young flies and preventing predation of the immature stages. During the wet season dung beetles may remove surface faeces so quickly that flies may have to compete for a severely limited resource resulting in higher numbers of flies per stool with density-dependent effects coming into play and further reducing the number of flies emerging. Perhaps most importantly we are uncertain whether flies carry *Chlamydia* on the surface of the fly or internally. If the pathogen is found largely on the body of a fly it will be more vulnerable to the effects of desiccation, whilst if carried internally it will be most sensitive to changes in temperature. Whilst many of these suggestions are conjectures, they do illustrate the potential complexity of the system and how difficult it is to predict how climate factors influence the risk of trachoma transmission.

More data on how climatic variables influence the current abundance of *M. sorbens* will allow forecasts to be made concerning the potential impact of future climate change, indicating areas where the role of *M. sorbens* as a vector of trachoma may vary in importance over time.

## Additional file

Additional file 1: Table S1. Search Terms for Systematic Literature Review. (DOCX 13 kb)
