# Supplementary material for: The impact of climate on the abundance of Musca sorbens, the vector of trachoma
Source: Parasit Vectors. 2016 Jan 27;9:48. doi: 10.1186/s13071-016-1330-y (PMC4730668; doi:10.1186/s13071-016-1330-y)
Supplement: Additional file 1: Table S1. — Search Terms for Systematic Literature Review. (DOCX 13 kb) [file 13071_2016_1330_MOESM1_ESM.docx]

**Supplementary Table S1. Search Terms for Systematic Literature Review**

1. fly/

2. house fly/

3. Muscidae/

4. Musca sorbens.mp.

5. sorbens.mp.

6. humilis.mp.

7. vetustissima.mp.

8. 1 or 2 or 3 or 4 or 5 or 6 or 7

9. Climate/

10. climate.mp.

11. climat$.mp.

12. Tropical Climate/ or Desert Climate/ or Cold Climate/

13. climate change.mp. or Climate Change/

14. Temperature/

15. temperature.mp.

16. Hot Temperature/ or Cold Temperature/

17. Rain/

18. rain$.mp.

19. precipitation.mp.

20. Disasters/ or Floods/

21. flood$.mp.

22. meteorologic$.mp.

23. Weather/ or Meteorological Concepts/ or Seasons/ or Humidity/

24. weather.mp.

25. season$.mp.

26. humid$.mp.

27. cold.mp.

28. heat$.mp.

29. heatwave$.mp.

30. heat wave$.mp.

31. dryland$.mp.

32. Droughts/

33. drought$.mp.

34. Cyclonic Storms/

35. cyclone$.mp.

36. typhoon$.mp.

37. hurricane$.mp.

38. monsoon$.mp.

39. tropical storm$.mp.

40. El Nino-Southern Oscillation/

41. El Nino.mp.

42. ENSO.mp.

43. southern oscillation.mp.

44. SOI.mp.

45. Wind/

46. wind$.mp.

47. elevation.mp. or geographic elevation/

48. altitude.mp. or altitude/

49. arid*.mp.

50. semiarid climate/ or desert climate/ or desert/ or ecosystem/

51. sahel.mp.

52. savanna/ or savanna*.mp.

53. eco* zone*.mp.

54. ecotone*.mp.

55. ecology/ or ecosystem/

56. ecologic*.mp.

57. 9 or 10 or 11 or 12 or 13 or 14 or 15 or 16 or 17 or 18 or 19 or 20 or 21 or 22 or 23 or 24 or 25 or 26 or 27 or 28 or 29 or 30 or 31 or 32 or 33 or 34 or 35 or 36 or 37 or 38 or 39 or 40 or 41 or 42 or 43 or 44 or 45 or 46 or 47 or 48 or 49 or 50 or 51 or 52 or 53 or 54 or 55 or 56

58. 8 and 57
